# Circulating arginine–nitric oxide pathway metabolites and nitrated fatty acids in relation to pathological features of colorectal cancer

**DOI:** 10.3389/fmolb.2026.1910147

**Published:** 2026-09-29

**Authors:** Jakub Klekowski, Paulina Fortuna, Łukasz Lewandowski, Gabriela Maciejewska, Wioleta Szewczak, Karolina Mosna, Marek Zawadzki, Mariusz Chabowski, Mariusz Fleszar

**Affiliations:** 1 Department of Surgery, 4th Military Clinical Hospital, Wroclaw, Poland; 2 Division of Anesthetic and Surgical Nursing, Department of Nursing, Faculty of Nursing and Midwifery, Wroclaw Medical University, Wroclaw, Poland; 3 Omics Research Center, Wroclaw Medical University, Wroclaw, Poland; 4 Department of Biochemistry and Immunochemistry, Wroclaw Medical University, Wroclaw, Poland; 5 Department of Clinical Surgical Sciences, Faculty of Medicine, Wroclaw University of Science and Technology, Wroclaw, Poland; 6 Department of Oncological Surgery, Research and Development Centre at Regional Specialist Hospital, Wroclaw, Poland

**Keywords:** arginine metabolism, colorectal cancer, fatty acids, metabolomics, nitric oxide

## Abstract

**Introduction:**

Colorectal cancer (CRC) is associated with metabolic and inflammatory disturbances, including alterations in the arginine–nitric oxide (NO) pathway. This study assessed serum metabolites related to the arginine–NO–nitrated fatty acid (NO₂-FA) axis in patients with CRC and examined their associations with tumor stage and pathological features.

**Methods:**

A total of 122 patients undergoing colorectal resection for CRC were prospectively enrolled, of whom 117 had complete measurements for the candidate-metabolite panel and were included in the analyses. Preoperative serum samples were analyzed using targeted LC-MS/MS. Associations between metabolite concentrations and tumor stage were evaluated using complementary exploratory modelling approaches, including ordinal regression for TNM stage, tumor extent (T), and nodal involvement (N), as well as binary logistic regression for advanced disease (TNM III–IV), angioinvasion, and neuroinvasion.

**Results:**

Among the analyzed metabolites, SDMA showed the most recurrent inverse association with pathological disease stage in the initial models. However, these associations attenuated after adjustment for age and sex and were further weakened after additional adjustment for serum creatinine, with all fully adjusted 95% confidence intervals (CIs) including unity. Thus, the observed SDMA pattern was not independent of renal function and should be regarded as hypothesis-generating rather than as a convincing stage-associated metabolic signal. In a complementary binary analysis of TNM stages III–IV versus I–II, an inverse association with SDMA persisted after adjustment only under the stepwise selection strategy. Lower agmatine concentrations were associated with higher T category under the elastic-net strategy. Under the stepwise strategy, higher ADMA concentrations were associated with lower odds of angioinvasion and neuroinvasion, whereas higher citrulline concentrations were associated with higher odds of neuroinvasion. These invasive-feature associations were not reproduced under the elastic-net strategy and should therefore be considered procedure-dependent exploratory findings. No clear association was detected between circulating nitrooleates and the analyzed pathological outcomes.

**Discussion:**

These exploratory findings identify preliminary stage-associated metabolic patterns within patients with CRC and require validation in independent cohorts.

## 1 Introduction

Colorectal cancer (CRC) is the third most common malignancy and the second leading cause of cancer-related deaths worldwide. Approximately 1.93 million new CRC cases and 940,000 CRC-related deaths occur worldwide annually. CRC incidence is higher in high-income regions, although the number of cases has also increased substantially in low- and middle-income countries, partly reflecting a shift towards more Westernized lifestyles and dietary patterns. The number of diagnosed adults under age of 50 is increasing. All the aforementioned features highlight the need for early detection and screening, but also novel diagnostic measures (Xi and Xu, 2021; Siegel et al., 2020a; Siegel et al., 2020b; Sung et al., 2021; Dekker et al., 2019).

There is an imbalance in regulating the inflammatory response in the tumor microenvironment in favor of the pro-inflammatory factors (Soundararajan et al., 2024; Mandal, 2018).

Nitric oxide (NO) has a concentration- and context-dependent dual role in cancer, with both tumor-promoting and tumor-suppressive effects reported (Vanini et al., 2015; Somasundaram et al., 2019). NO is synthesized by three nitric oxide synthase (NOS) isoforms: neuronal NOS (nNOS/NOS1), endothelial NOS (eNOS/NOS3), and inducible NOS (iNOS/NOS2). Whereas nNOS and eNOS generally generate lower amounts of NO over shorter periods, iNOS can produce substantially higher and more sustained NO levels (Vanini et al., 2015). The biological effects of NO in cancer depend not only on its concentration but also on the duration and spatial distribution of NO production, the cellular source, and the local tumor microenvironment (Vanini et al., 2015; Somasundaram et al., 2019). Consequently, NO may support signaling processes associated with tumor progression under some conditions, while higher or sustained NO exposure can induce cytotoxic and antitumor responses (Vanini et al., 2015; Somasundaram et al., 2019).

Nitrated fatty acids (NO_2_-FA) are formed through the reaction of unsaturated fatty acids (UFAs) such as linolenic and oleic acid with secondary metabolites of NO or nitrogen dioxide anions (NO_2_-). The field of nitrated lipids is a rather novel subject of interest for scientists, with an increasing research intensity for the last 10–15 years. NO_2_-FAs are the best described products of reactive nitrogen species and have been found in tissues and biofluids. They were associated with several biological effects, especially anti-inflammatory function. The alkene group and nitro-alkene moiety of NO_2_-FA are responsible for the distinct biological activity and enable the covalent link (via Michael addition) to proteins. NO_2_-FAs are capable of reacting with cysteine thiol groups, thereby altering protein functions and contributing to the reduction of inflammatory responses - primarily through pathways involving PPAR-γ, NF-κB, and lipoxygenases. Derivatives of NO_2_-FAs have demonstrated cytotoxic effects in a study involving CRC cell lines (Melo et al., 2019; Koutoulogenis and Kokotos, 2021; Batthyany et al., 2006; Kühn et al., 2018).Arginine is a central substrate for several metabolic pathways relevant to CRC, including NO production through NOS and polyamine biosynthesis following its conversion to ornithine. Alterations in arginine transport and metabolism have been described in CRC, but their biological consequences are context-dependent (Du and Han, 2021; Alexandrou et al., 2018). Experimental studies have investigated arginine restriction or deprivation as a potential therapeutic strategy; however, sensitivity varies according to tumor metabolic characteristics, and the available evidence does not establish that dietary arginine reduction lowers the risk of developing CRC (Du and Han, 2021; Alexandrou et al., 2018).

Arginine undergoes post-translational modification (PTM) and its methylation is common in all eukaryotic organisms. The further downstream modification leads to production of asymmetric dimethylarginine (ADMA), which is an endogenous NOS inhibitor or symmetric dimethylarginine (SDMA) which decreases L-arginine transport through cellular membrane by competition (Fulton et al., 2019; Brankovic et al., 2017). Given the biological roles of NO and its endogenous methylated arginine derivatives, ADMA and SDMA represent relevant circulating metabolites for investigating alterations within the arginine–NO axis. Accordingly, the aim of the present study was to quantify selected circulating metabolites related to the arginine–NO pathway and nitrated fatty acids and to evaluate their associations with pathological stage and invasive tumour features in patients with CRC. The analyses were intended to generate hypotheses regarding stage-associated metabolic patterns rather than to establish disease-specific biomarkers or molecular mechanisms.

## 2 Materials and methods

### 2.1 Studied group

The studied group was prospectively recruited among patients qualified for colorectal resections due to CRC in the 4th Military Clinical Hospital in Wroclaw, Poland. All patients participating in the study gave their written consent to be included. Anthropometric, demographic, and clinical data were collected based on patients’ medical history. The blood samples were collected from every patient prior to the surgical procedure.

Blood was collected into serum-separating tubes (SST, clot activator), allowed to clot for 30 min at room temperature, and centrifuged at 1,500 *g* for 10 min in the hospital laboratory. Serum was carefully aspirated within 45–60 min from venipuncture, aliquoted, and immediately stored at −80 °C. All samples remained unthawed until the metabolomic workflow, resulting in zero freeze–thaw cycles. The total storage duration at −80 °C before analysis ranged from 6 to 24 months, depending on the date of sample collection.

Prior to LC-MS/MS analysis, samples were randomized to minimize batch effects. The analytical sequence included pooled QC (quality control) samples injected every 24 runs, solvent blanks, and matrix blanks. The dataset was acquired in two analytical batches, each containing an identical QC structure. Laboratory personnel performing sample preparation and LC-MS/MS acquisition were blinded to the pathological stage and clinical characteristics of the patients.

The detailed characteristics of the studied group are presented in Table 1. The final analysis included 117 subjects. More information on data completeness is shown in the Results, in its respective section. The studied group was derived from the same prospectively recruited CRC cohort described previously (Klekowski et al., 2026); however, the present study used an independently generated dataset obtained from the same serum samples through a distinct sample preparation protocol, targeted LC-MS/MS workflow, and analyte panel focused on arginine metabolism, nitric oxide-related intermediates, and nitrated fatty acids.

**TABLE 1:** Cohort characteristics.

Section	Characteristic	Level or statistic	TNM I–II (n = 63)	TNM III–IV (n = 54)	Notes
Demographic and anthropometric	Age, years	Median [Q1–Q3]	71.000 [66.000–77.500]	67.500 [57.500–74.000]	
	Sex	Female	34 (54.0%)	23 (42.6%)	
		Male	29 (46.0%)	31 (57.4%)	
	BMI, kg/m^2^	Median [Q1–Q3]	26.790 [23.695–28.300]	26.330 [22.312–29.317]	
Renal function	Serum creatinine, mg/dL	Median [Q1–Q3]	0.850 [0.755–1.065]	0.825 [0.720–0.920]	
	Chronic kidney disease	No	57 (90.5%)	53 (98.1%)	
		Yes	6 (9.5%)	1 (1.9%)	
Tumor characteristics	Tumor size, mm	Median [Q1–Q3]	40.000 [30.000–45.000]	40.000 [30.000–50.000]	
	CEA	Median [Q1–Q3]	2.490 [1.703–5.595]	3.560 [1.875–16.270]	{1}
	Histological subtype	Adenocarcinoma	51 (81.0%)	45 (83.3%)	
		Mucinous adenocarcinoma or adenocarcinoma with a mucinous component	12 (19.0%)	8 (14.8%)	
		Complete pathological response	0 (0.0%)	1 (1.9%)	
	Tumor location	Cecum	9 (14.3%)	9 (16.7%)	
		Ascending colon	10 (15.9%)	9 (16.7%)	
		Hepatic flexure	3 (4.8%)	3 (5.6%)	
		Transverse colon	2 (3.2%)	0 (0.0%)	
		Splenic flexure	1 (1.6%)	0 (0.0%)	
		Descending colon	1 (1.6%)	1 (1.9%)	
		Sigmoid colon	23 (36.5%)	20 (37.0%)	
		Rectum	14 (22.2%)	12 (22.2%)	
	Histological grade	G1	31 (49.2%)	22 (40.7%)	
		G2	29 (46.0%)	30 (55.6%)	
		G3	3 (4.8%)	2 (3.7%)	
	TNM stage	I	15 (23.8%)	0 (0.0%)	
		II	48 (76.2%)	0 (0.0%)	
		III	0 (0.0%)	39 (72.2%)	
		IV	0 (0.0%)	15 (27.8%)	
	T category	T0	0 (0.0%)	1 (1.9%)	
		T1	8 (12.7%)	0 (0.0%)	
		T2	7 (11.1%)	5 (9.3%)	
		T3	37 (58.7%)	27 (50.0%)	
		T4a	7 (11.1%)	19 (35.2%)	
		T4b	4 (6.3%)	2 (3.7%)	
	N category	N0	63 (100.0%)	2 (3.7%)	
		N1	0 (0.0%)	33 (61.1%)	
		N2	0 (0.0%)	19 (35.2%)	
	M category	M0	63 (100.0%)	39 (72.2%)	
		M1	0 (0.0%)	15 (27.8%)	
	Angioinvasion	Absent	45 (71.4%)	12 (22.2%)	
		Present	18 (28.6%)	42 (77.8%)	
	Neuroinvasion	Absent	54 (85.7%)	33 (61.1%)	
		Present	9 (14.3%)	21 (38.9%)	
Neoadjuvant treatment	Preoperative radiotherapy	No	55 (87.3%)	49 (90.7%)	
		Yes	8 (12.7%)	5 (9.3%)	
	Preoperative chemotherapy	No	60 (95.2%)	50 (92.6%)	
		Yes	3 (4.8%)	4 (7.4%)	
Comorbidities	Diabetes mellitus	No	45 (71.4%)	44 (81.5%)	
		Type 2 diabetes	18 (28.6%)	10 (18.5%)	
	Hypertension	No	18 (28.6%)	23 (42.6%)	
		Yes	45 (71.4%)	31 (57.4%)	
	Heart failure	No	53 (84.1%)	51 (94.4%)	
		Yes	10 (15.9%)	3 (5.6%)	
	Ischemic heart disease	No	50 (79.4%)	46 (85.2%)	
		Yes	13 (20.6%)	8 (14.8%)	
	Arrhythmia	No	50 (79.4%)	43 (79.6%)	
		Yes	13 (20.6%)	11 (20.4%)	
	Hyperlipidemia	No	45 (71.4%)	38 (70.4%)	
		Yes	18 (28.6%)	16 (29.6%)	
	Asthma	No	60 (95.2%)	52 (96.3%)	
		Yes	3 (4.8%)	2 (3.7%)	
	COPD	No	60 (95.2%)	51 (94.4%)	
		Yes	3 (4.8%)	3 (5.6%)	
	Thyroid disease	None	55 (87.3%)	52 (96.3%)	
		Hypothyroidism	7 (11.1%)	1 (1.9%)	
		Hyperthyroidism	1 (1.6%)	1 (1.9%)	
	Liver disease	No	62 (98.4%)	54 (100.0%)	
		Yes	1 (1.6%)	0 (0.0%)	
Smoking	Smoking status	Never smoker	37 (58.7%)	31 (57.4%)	
		Current smoker	2 (3.2%)	3 (5.6%)	
		Former smoker	24 (38.1%)	20 (37.0%)	
	Smoking exposure, pack-years	Median [Q1–Q3] among ever-smokers	30.000 [16.500–40.000]	25.000 [10.000–40.000]	{2}
Medication use	Acetylsalicylic acid	No	56 (88.9%)	48 (88.9%)	
		Yes	7 (11.1%)	6 (11.1%)	
	Metformin	No	42 (66.7%)	43 (79.6%)	
		Yes	21 (33.3%)	11 (20.4%)	
	ACE inhibitors	No	40 (63.5%)	31 (57.4%)	
		Yes	23 (36.5%)	23 (42.6%)	
	Angiotensin receptor blockers	No	53 (84.1%)	50 (92.6%)	
		Yes	10 (15.9%)	4 (7.4%)	
	Statins	No	32 (50.8%)	34 (63.0%)	
		Yes	31 (49.2%)	20 (37.0%)	
	Ezetimibe	No	60 (95.2%)	51 (94.4%)	
		Yes	3 (4.8%)	3 (5.6%)	
	Beta-blockers	No	35 (55.6%)	30 (55.6%)	
		Yes	28 (44.4%)	24 (44.4%)	
	Alpha-blockers	No	54 (85.7%)	48 (88.9%)	
		Yes	9 (14.3%)	6 (11.1%)	

{1} Available n: 58 and 51, respectively; {2} Available n: 24 and 22, respectively.

### 2.2 Materials

Analytical standards of 10-Nitrooleate and 9-Nitrooleate, together with isotope-labeled internal standards (9-Nitrooleate-d17 and 10-Nitrooleate-d17), were obtained from Cayman Chemical (Ann Arbor, MI, United States), Hydrochloride salts of unlabelled dimethylamine (DMA), hexadeutero-dimethylamine (D6-DMA, declared as 99 atom % 2H), L-arginine, SDMA, ADMA, L-citrulline, L-Ornithine monohydrochloride, labelled L-Ornithine hydrochloride (3,3,4,4,5,5-D6-ornithine), putrescine, spermidine, spermine, agmatine, 1,4-Diaminobutane-2,2,3,3-d4 dihydrochloride, spermidine-(butyl-d8) trihydrochloride, spermine-(butyl-d8) tetrahydrochloride, benzoyl chloride (BCl) and sodium tetraborate were procured from Sigma-Aldrich (Poznan, Poland). Isotope-labelled L-arginine:HCl (D7-arginine, 98%) and asymmetric dimethylarginine (2,3,3,4,4,5,5-D7-ADMA, 98%) were obtained from Cambridge Isotope Laboratories (Tewksbury, MA, United States). Methanol, water, ethanol, acetonitrile (ACN), ammonium formate, and formic acid were purchased from Witko (Warsaw, Poland).

### 2.3 Standards and sample preparation

#### 2.3.1 Nitro fatty acids

Samples were subjected to quantitative targeted metabolomic analysis using LC-MS/MS. Briefly, 50 µL of serum samples or calibration standards (in the range from 0.0125 to 1.0 nM) were transferred into 2 mL Eppendorf tubes. Subsequently, 10 µL of 0.1% butylated hydroxytoluene (BHT) in ethanol and 10 µL of internal standard solution were added. The mixtures were vortex-mixed for 1 min. Next, 300 µL of ethanol containing 0.1% formic acid was added, followed by mixing for 10 min at room temperature. Samples were then centrifuged, and 250 µL of the obtained supernatant was collected and dried. The dried extracts were reconstituted in 60 µL of 75% ethanol containing 0.1% formic acid prior to LC-MS/MS analysis.

Chromatographic separation was performed using an ACQUITY UPLC system coupled to a Xevo TQ Absolute triple quadrupole mass spectrometer (Waters, Milford, MA, United States). Separation was achieved on a BEH C18 analytical column maintained at 50 °C. The mobile phase consisted of 10 mM ammonium formate with 0.1% formic acid (solvent A) and acetonitrile (solvent B). The gradient elution was performed at a flow rate of 0.25 mL/min as follows: 20% B (0–2.0 min), 45% B (2.5 min), 75% B (3.5 min), 95% B (4.5–8.5 min), followed by re-equilibration to initial conditions until 10.6 min.

Mass spectrometric detection was carried out in negative electrospray ionization (ESI) mode with the following source parameters: capillary voltage 2.0 kV, source temperature 150 °C, desolvation temperature 400 °C, cone gas flow 150 L/h, and desolvation gas flow 800 L/h. Quantitative analysis was performed in multiple reaction monitoring (MRM) mode using optimized transitions for nitro-fatty acids and isotope-labelled internal standards.

The analytical performance of the nitrooleate assay was evaluated separately for 9-nitrooleic acid and 10-nitrooleic acid. The limits of detection were 0.0076 nM and 0.0036 nM for 9-nitrooleate and 10-nitrooleate, respectively, and the lower limit of quantification (LLOQ) was 0.0125 nM for both analytes. Analytical precision was evaluated at 0.0125, 0.25, and 1.0 nM. For 9-nitrooleate, intra-day CVs were 23.89%, 13.71%, and 7.05%, respectively, whereas the corresponding inter-day CVs were 15.24%, 13.71%, and 7.31%. For 10-nitrooleate, intra-day CVs were 24.49%, 4.36%, and 4.83%, and inter-day CVs were 24.76%, 9.51%, and 9.44%, respectively. Thus, imprecision exceeding 20% was confined to the lowest tested concentration level, whereas both intra- and inter-day CVs were below 15% at 0.25 and 1.0 nM for both analytes.

The influence of the serum matrix on analyte recovery was assessed using serum samples spiked at the same concentration levels. Apparent recoveries for 9-nitrooleate were 82.87%, 86.58%, and 108.55% at 0.0125, 0.25, and 1.0 nM, respectively, whereas the corresponding values for 10-nitrooleate were 82.10%, 86.19%, and 110.18%.

Calibration performance was evaluated separately for each analyte using unweighted ordinary least-squares linear regression of analytically determined concentrations against nominal concentrations. For 9-nitrooleate, the regression equation was y = 0.0301 + 1.8297x (R^2^ = 0.8388), whereas for 10-nitrooleate the equation was y = 0.0545 + 0.9102x (R^2^ = 0.9536).

For reference, analytical performance was interpreted against the ICH M10 criteria for chromatographic bioanalytical methods, which recommend precision of ≤15% CV at concentrations above the LLOQ and ≤20% CV at the LLOQ. Because the present assay was applied in an exploratory biomarker study rather than regulated drug bioanalysis, these criteria were used as a reference framework rather than as evidence of full regulatory validation.

In the final analytical cohort, 9 of 117 measurements (7.7%) for 9-nitrooleate were below the LOQ, whereas no measurements for 10-nitrooleate were below the LOQ. For statistical analyses, concentrations below the LOQ were assigned a value of 0.00125 nM, corresponding to one-tenth of the LOQ.

#### 2.3.2 Nitric oxide pathway intermediates

The extraction and derivatization of metabolites associated with Arg metabolic pathways were carried out using the previously described method (Fl et al., 2018). One hundred microliters of calibration standards or serum samples were mixed with 50 µL of borate buffer (pH = 9.2) and 10 µL of internal standard solution of D6-arginine, D6-DMA, D7-ADMA, D6-ornithine, 1,4-Diaminobutane-2,2,3,3-d4, spermidine-(butyl-d8), spermine-(butyl-d8) (100 μM, 50 μM, 20 μM, 100 μM, 70 μM, 100 μM, 100 μM, 100 μM, respectively). After mixing, 400 µL of acetonitrile and 10 µL of 10% BCl in acetonitrile were added. Then, the mixture was vortexed for 10 min at 25 °C. The samples were centrifuged at 10,000 rpm for 7 min at 4 °C. Obtained supernatants were diluted 4:1 with water.

Chromatographic separation was performed using an ACQUITY UPLC system coupled to a Xevo TQ Absolute triple quadrupole mass spectrometer (Waters, Milford, MA, United States). Separation was achieved on a BEH ShieldRP18 analytical column maintained at 60 °C. The mobile phase consisted of water with 0.1% formic acid (solvent A) and methanol with 0.1%FA (solvent B). The gradient elution was performed at a flow rate of 0.30 mL/min as follows: 3.0% B (0–1.5 min), 15% B (3.5 min), 40% B (5.0 min), 90% B (7.5–10.5 min), followed by re-equilibration to initial conditions until 12.5 min.

Mass spectrometric detection was carried out in positive electrospray ionization (ESI) mode with the following source parameters: capillary voltage 2.0 kV, source temperature 150 °C, desolvation temperature 600 °C, cone gas flow 150 L/h, and desolvation gas flow 800 L/h. Quantitative analysis was performed in multiple reaction monitoring (MRM) mode using optimized transitions.

### 2.4 Statistical analysis

#### 2.4.1 Preprocessing and outcome definition

Continuous metabolite concentrations were considered candidate explanatory variables in relation to tumour stage and pathological features. To reduce the influence of outliers and scale heterogeneity, all metabolite variables were robustly standardised using the median and interquartile range prior to modelling; clinical covariates were left on their natural scale. Tumour stage was analysed using ordinal regression models, treating TNM stage as an ordered categorical outcome. In addition, individual components of tumour burden were analysed separately to improve biological interpretability, including tumour extent category (T), nodal involvement (N), and the presence of angioinvasion or neuroinvasion. For selected analyses, TNM stage was additionally dichotomised into non-advanced (TNM I-II) versus advanced disease (TNM III-IV) and analysed using binary logistic regression. From the mentioned outcomes, TNM was considered primary.

To reduce sparse-category issues and improve model stability, selected outcome categories were combined *a priori* based on low cell counts and clinical similarity. Specifically:

- Tumor extent (T category):
  ◦ Subcategories T4a and T4b were collapsed into a single T4 category.
  ◦ In addition, rare observations originally coded as T0 were merged with T1, resulting in a four-level ordered tumor size variable (T1–T4) used in all analyses. This decision was motivated by the very low frequency of T0 observations and their limited clinical interpretability in the present dataset.
- Nodal involvement (N category):
  ◦ Subcategories N1a, N1b, and N1c were collapsed into a unified N1 category.
  ◦ Subcategories N2a and N2b were collapsed into a unified N2 category.
  ◦ The final nodal outcome was analyzed as a three-level ordered variable (N0–N2).
- Overall TNM stage:
  ◦ TNM stage was analyzed primarily as an ordered categorical variable.
  ◦ For secondary analyses, TNM stage was dichotomized into non-advanced versus advanced disease, with advanced disease defined as TNM stage III and IV, and non-advanced disease as TNM I and II.

All collapsed outcomes were treated as ordered factors in ordinal regression models.

#### 2.4.2 Modelling strategy

Two complementary exploratory variable-selection strategies were applied separately for each outcome. Twelve metabolite concentrations were considered as candidate explanatory variables. In Strategy A, elastic-net penalized regression was used for variable selection. The elastic-net mixing parameter was fixed at α = 0.75 to provide a lasso-dominant penalty favoring sparse selection while retaining a smaller ridge component for stabilization in the presence of correlated metabolites. The regularization parameter λ was selected by five-fold cross-validation using the one-standard-error rule. The value of α was not optimized for predictive performance. Cumulative-logit elastic-net models were used for ordinal outcomes, whereas elastic-net logistic regression was used for binary outcomes. To restrict model complexity in relation to the available sample size, a maximum of four metabolites was retained in each baseline model. If more than four metabolites had non-zero penalized coefficients at the selected λ, metabolites were ranked according to the absolute magnitude of their penalized coefficients and the four highest-ranked variables were retained. This ceiling was not intended to represent a statistically optimal model size. As all metabolites had been scaled by their median and interquartile range, variables exceeding the ceiling could be ranked on a common scale according to the absolute magnitude of their penalized coefficients. The selected metabolites were subsequently refitted in unpenalized regression models to obtain odds ratios (ORs) and 95% CIs. In Strategy B, a bidirectional stepwise procedure based on likelihood-ratio tests was used as a sensitivity analysis. Candidate metabolites entered the model at p < 0.05 and were removed at p ≥ 0.10.

For both strategies, M0 denoted a metabolite-only model, whereas M1 additionally included age and sex. These covariates were included irrespective of their statistical significance. The two selection strategies were used to examine the dependence of the findings on the modelling procedure rather than as independent confirmatory analyses. Metabolites retained by only one strategy were therefore considered strategy-dependent and interpreted with additional caution. Age and sex were included as common covariates to provide a limited and consistent adjustment across outcomes; the M1 specification was not intended to represent a complete causal adjustment set. In an additional fixed-model sensitivity analysis, serum creatinine was added to the final models containing SDMA or ADMA because renal handling is an important determinant of circulating dimethylarginines. Serum creatinine was available for all 117 participants in the analytical cohort. Variable selection was not repeated in this sensitivity analysis. Other potential determinants of circulating metabolite concentrations, including inflammatory and nutritional status, body composition, comorbidities, medication use, dietary factors, and tumor location, were not comprehensively available and could not be incorporated into consistently specified multivariable models.

Ordinal outcomes were analyzed using cumulative-logit models under the proportional-odds assumption. Positive regression coefficients corresponded to greater odds of belonging to a higher outcome category. The proportional-odds assumption was assessed using the Brant test. The proportional-odds assumption was assessed after variable selection for the final unpenalized M0 and M1 ordinal-model specifications. Brant tests were calculated using equivalent proportional-odds models fitted to the same analytical cohorts. Complete omnibus and predictor-specific results are provided in Supplementary Files 1, 2. Binary outcomes were analyzed using logistic regression. ORs for metabolites were expressed per one-interquartile-range increase in concentration following robust scaling by the median and interquartile range. Metabolite effects were modelled as linear terms on the log-odds scale; spline functions or other nonlinear terms were not fitted.

No formal correction for multiple comparisons was applied. The analyses combined data-driven variable selection with subsequent unpenalized model refitting across several related pathological outcomes. Consequently, the reported p-values and CIs do not account for the full uncertainty introduced by variable selection or outcome multiplicity and should be interpreted descriptively. The two modelling strategies were used to assess the dependence of the findings on the selection procedure rather than as independent confirmatory analyses. All findings were therefore considered exploratory and hypothesis-generating and require confirmation in an independent cohort.

Likelihood ratio tests were used to compare nested models. All analyses were conducted using complete-case data for the variables included in each model. Model outputs with their associated README file are given in Supplementary Materials. The unpenalized refitted coefficients and CIs should be interpreted as descriptive estimates conditional on the models selected in the present cohort and not as selection-adjusted inferential estimates. All reported p-values are nominal post-selection values provided for descriptive purposes. No result is interpreted as a multiplicity-controlled statistical discovery.

Only one observation was originally classified as T0. In the primary analysis, this observation was combined with T1 to avoid retaining a single-observation ordinal category. In a *post hoc* sensitivity analysis, the T0 observation was excluded and both metabolite-selection strategies were rerun. For the elastic-net analysis, the original cross-validation fold membership was preserved for all participants common to the primary and sensitivity-analysis cohorts.

As a *post hoc* small-sample sensitivity analysis for neuroinvasion, the fixed M0 and M1 specifications obtained under Strategies A and B were refitted using Firth bias-reduced logistic regression without repeating variable selection. For models containing predictors, 95% CIs were obtained from the profile penalized likelihood and p-values from penalized likelihood-ratio tests. A Wald interval and p-value were used only for the intercept-only Strategy A M0 specification. This analysis assessed whether coefficient estimates in the fixed models were materially affected by first-order small-sample bias or possible separation; it did not assess predictor-selection stability, post-selection uncertainty, or predictive performance.

## 3 Results

### 3.1 Multivariate modelling according to both strategies

#### 3.1.1 Primary outcome: TNM stage

For ordinal TNM stage, SDMA was the only metabolite retained by both variable-selection strategies. In the metabolite-only models, higher SDMA concentrations were associated with lower odds of belonging to a higher TNM category. After adjustment for age and sex, the estimated ORs remained below 1 but moved towards the null, and the 95% CIs included 1 in both strategies. DMA, spermidine, and 10-nitrooleate were additionally retained under Strategy A, but their estimates were imprecise and showed no clear evidence of association with TNM stage.

In the complementary binary analysis comparing TNM stages III–IV with stages I–II, no metabolite was retained under Strategy A. Under Strategy B, SDMA was retained in both M0 and M1, with higher concentrations associated with lower odds of TNM stage III–IV. This finding was therefore specific to the stepwise selection strategy. The results are shown in Table 2 and visualized in Figure 1.

**TABLE 2:** Modelling the primary outcome—TNM.

Metabolite	Values used for centering		Strategy A: unadjusted (M0)		Strategy A: adjusted (M1)		Strategy B: unadjusted (M0)		Strategy B: adjusted (M1)	
	Median	IQR	OR (95% CI)	p	OR (95% CI)	p	OR (95% CI)	p	OR (95% CI)	p
Modelled: TNM (ordinal)										
ADMA	0.383	0.107	—	—	—	—	—	—	—	—
Agmatine	0.057	0.018	—	—	—	—	—	—	—	—
L-Arginine	21.761	8.645	—	—	—	—	—	—	—	—
L-Citrulline	21.003	11.797	—	—	—	—	—	—	—	—
DMA	0.032	0.015	0.98 (0.93–1.04)	0.502	0.98 (0.93–1.03)	0.426	—	—	—	—
L-Ornithine	42.702	18.637	—	—	—	—	—	—	—	—
Putrescine	0.113	0.096	—	—	—	—	—	—	—	—
Spermidine	0.083	0.042	0.95 (0.83–1.09)	0.451	0.95 (0.83–1.09)	0.461	—	—	—	—
Spermine	0.150	0.023	—	—	—	—	—	—	—	—
SDMA	0.385	0.126	0.67 (0.47–0.95)	0.023	0.76 (0.51–1.12)	0.167	0.65 (0.46–0.92)	0.016	0.71 (0.48–1.05)	0.083
9-nitrooleate	0.655	1.800	—	—	—	—	—	—	—	—
10-nitrooleate	0.342	0.495	1.05 (0.97–1.14)	0.225	1.06 (0.97–1.16)	0.212	—	—	—	—
Modelled: TNM ≥3 (binomial)										
ADMA	0.383	0.107	—	—	—	—	—	—	—	—
Agmatine	0.057	0.018	—	—	—	—	—	—	—	—
L-Arginine	21.761	8.645	—	—	—	—	—	—	—	—
L-Citrulline	21.003	11.797	—	—	—	—	—	—	—	—
DMA	0.032	0.015	—	—	—	—	—	—	—	—
L-Ornithine	42.702	18.637	—	—	—	—	—	—	—	—
Putrescine	0.113	0.096	—	—	—	—	—	—	—	—
Spermidine	0.083	0.042	—	—	—	—	—	—	—	—
Spermine	0.150	0.023	—	—	—	—	—	—	—	—
SDMA	0.385	0.126	—	—	—	—	0.53 (0.34–0.83)	0.006	0.57 (0.35–0.94)	0.026
9-nitrooleate	0.655	1.800	—	—	—	—	—	—	—	—
10-nitrooleate	0.342	0.495	—	—	—	—	—	—	—	—

OR, expressed per 1 IQR, increase in metabolite concentration (robust-scaled). More information on the models is given in Supplementary Materials. All p-values are nominal and do not account for variable selection or outcome multiplicity. Details regarding scaling are shown in Supplementary Files 1, 2.

**FIGURE 1:**
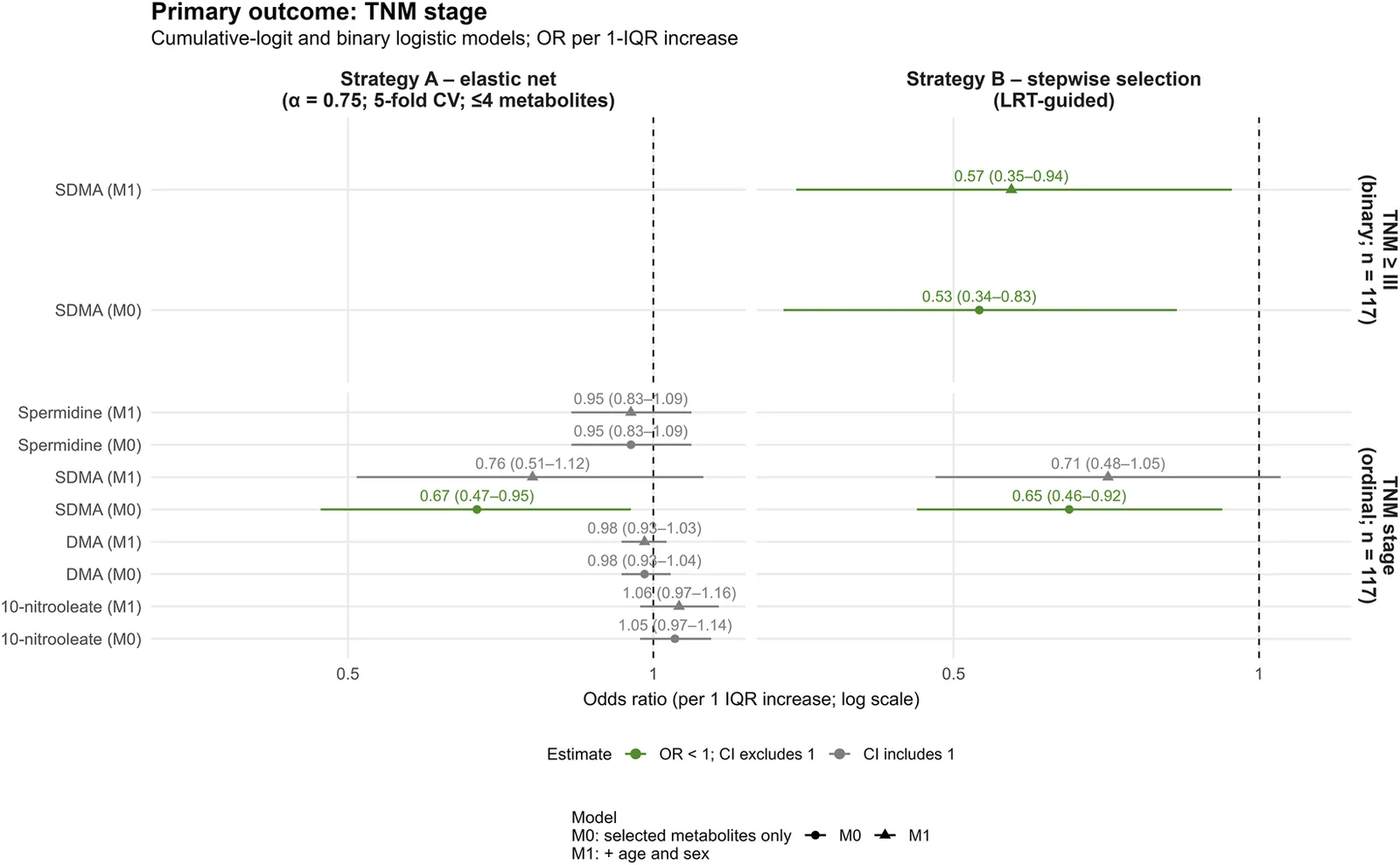
Associations of selected metabolites with TNM stage. Forest plots present ORs and 95% CIs for metabolites retained using Strategy A (elastic net; α = 0.75; five-fold cross-validation; one-standard-error rule; maximum of four retained metabolites) or Strategy B (likelihood-ratio-test-guided stepwise selection). Ordinal TNM stage was analyzed using cumulative-logit regression, whereas TNM stages III–IV versus I–II were analyzed using binary logistic regression. M0 included the retained metabolites; M1 was additionally adjusted for age and sex. ORs are reported per one-interquartile-range increase in metabolite concentration after robust scaling. All displayed ordinal and binary TNM models included 117 participants. CI, confidence interval; CV, cross-validation; IQR, interquartile range; LRT, likelihood-ratio test; TNM, tumor–node–metastasis.

#### 3.1.2 Secondary outcomes

Before application of the four-metabolite cap, the elastic-net procedure retained 11 metabolites for T category and eight for N category. The cap therefore affected these two outcomes. It did not alter selection for ordinal TNM, for which exactly four metabolites were retained, and was not invoked for binary TNM, angioinvasion, or neuroinvasion, for which no metabolite had a non-zero coefficient.

For ordinal T category, agmatine, ADMA, ornithine, and putrescine were retained under Strategy A, whereas no metabolite was retained under Strategy B. Among the Strategy A estimates, agmatine showed the clearest inverse association with higher T category, which changed little after adjustment for age and sex. Because agmatine was not retained by the alternative selection strategy, this finding was considered strategy-dependent.

For ordinal N category, SDMA was retained under both strategies and showed an inverse direction of association with higher nodal category. The effect estimates attenuated after adjustment for age and sex, and the adjusted 95% CIs included 1. Arginine, spermine, and 10-nitrooleate were additionally retained under Strategy A, but their estimates were imprecise and did not provide clear evidence of association.

For angioinvasion, ADMA was retained only under Strategy B, with higher ADMA concentrations associated with lower odds of angioinvasion in both the metabolite-only model and the model adjusted for age and sex. For neuroinvasion, Strategy B retained both ADMA and citrulline. In the joint age- and sex-adjusted model, higher ADMA concentrations were associated with lower odds of neuroinvasion (OR 0.36, 95% CI 0.17–0.76), whereas higher citrulline concentrations were associated with higher odds of neuroinvasion (OR 1.98, 95% CI 1.07–3.65). Similar estimates were observed in the metabolite-only model: OR 0.36 (95% CI 0.18–0.75) for ADMA and OR 1.80 (95% CI 1.002–3.25) for citrulline. Neither the angioinvasion finding nor the neuroinvasion findings were reproduced under Strategy A and should therefore be interpreted as strategy-dependent exploratory associations.

No clear association between circulating 9-nitrooleate or 10-nitrooleate and the analyzed pathological outcomes was identified across the modelling strategies. The results are shown in Table 3 and visualized in Figure 2.

**TABLE 3:** Modelling the secondary outcomes.

Metabolite	Values used for centering		Strategy A: unadjusted (M0)		Strategy A: adjusted (M1)		Strategy B: unadjusted (M0)		Strategy B: adjusted (M1)	
	Median	IQR	OR (95% CI)	p	OR (95% CI)	p	OR (95% CI)	p	OR (95% CI)	p
Modelled: T (ordinal)										
ADMA	0.383	0.107	1.34 (0.77–2.35)	0.299	1.35 (0.77–2.38)	0.293	—	—	—	—
Agmatine	0.057	0.018	0.46 (0.23–0.95)	0.034	0.46 (0.23–0.94)	0.034	—	—	—	—
L-Arginine	21.761	8.645	—	—	—	—	—	—	—	—
L-Citrulline	21.003	11.797	—	—	—	—	—	—	—	—
DMA	0.032	0.015	—	—	—	—	—	—	—	—
L-Ornithine	42.702	18.637	0.67 (0.42–1.08)	0.100	0.67 (0.42–1.08)	0.102	—	—	—	—
Putrescine	0.113	0.096	1.37 (0.80–2.37)	0.253	1.37 (0.79–2.37)	0.257	—	—	—	—
Spermidine	0.083	0.042	—	—	—	—	—	—	—	—
Spermine	0.150	0.023	—	—	—	—	—	—	—	—
SDMA	0.385	0.126	—	—	—	—	—	—	—	—
9-nitrooleate	0.655	1.800	—	—	—	—	—	—	—	—
10-nitrooleate	0.342	0.495	—	—	—	—	—	—	—	—
Modelled: N (ordinal)										
ADMA	0.383	0.107	—	—	—	—	—	—	—	—
Agmatine	0.057	0.018	—	—	—	—	—	—	—	—
L-Arginine	21.761	8.645	1.33 (0.87–2.02)	0.184	1.28 (0.84–1.96)	0.253	—	—	—	—
L-Citrulline	21.003	11.797	—	—	—	—	—	—	—	—
DMA	0.032	0.015	—	—	—	—	—	—	—	—
L-Ornithine	42.702	18.637	—	—	—	—	—	—	—	—
Putrescine	0.113	0.096	—	—	—	—	—	—	—	—
Spermidine	0.083	0.042	—	—	—	—	—	—	—	—
Spermine	0.150	0.023	0.94 (0.84–1.05)	0.269	0.93 (0.83–1.05)	0.248	—	—	—	—
SDMA	0.385	0.126	0.61 (0.40–0.93)	0.022	0.69 (0.43–1.09)	0.114	0.61 (0.40–0.93)	0.021	0.67 (0.43–1.05)	0.080
9-nitrooleate	0.655	1.800	—	—	—	—	—	—	—	—
10-nitrooleate	0.342	0.495	1.04 (0.97–1.13)	0.256	1.05 (0.97–1.14)	0.231	—	—	—	—
Modelled: Angioinvasion (binomial)										
ADMA	0.383	0.107	—	—	—	—	0.51 (0.31–0.86)	0.011	0.52 (0.31–0.87)	0.012
Agmatine	0.057	0.018	—	—	—	—	—	—	—	—
L-Arginine	21.761	8.645	—	—	—	—	—	—	—	—
L-Citrulline	21.003	11.797	—	—	—	—	—	—	—	—
DMA	0.032	0.015	—	—	—	—	—	—	—	—
L-Ornithine	42.702	18.637	—	—	—	—	—	—	—	—
Putrescine	0.113	0.096	—	—	—	—	—	—	—	—
Spermidine	0.083	0.042	—	—	—	—	—	—	—	—
Spermine	0.150	0.023	—	—	—	—	—	—	—	—
SDMA	0.385	0.126	—	—	—	—	—	—	—	—
9-nitrooleate	0.655	1.800	—	—	—	—	—	—	—	—
10-nitrooleate	0.342	0.495	—	—	—	—	—	—	—	—
Modelled: Neuroinvasion (binomial)										
ADMA	0.383	0.107	—	—	—	—	0.36 (0.18–0.75)	0.006	0.36 (0.17–0.76)	0.007
Agmatine	0.057	0.018	—	—	—	—	—	—	—	—
L-Arginine	21.761	8.645	—	—	—	—	—	—	—	—
L-Citrulline	21.003	11.797	—	—	—	—	1.80 (1.002–3.25)	0.049	1.98 (1.07–3.65)	0.029
DMA	0.032	0.015	—	—	—	—	—	—	—	—
L-Ornithine	42.702	18.637	—	—	—	—	—	—	—	—
Putrescine	0.113	0.096	—	—	—	—	—	—	—	—
Spermidine	0.083	0.042	—	—	—	—	—	—	—	—
Spermine	0.150	0.023	—	—	—	—	—	—	—	—
SDMA	0.385	0.126	—	—	—	—	—	—	—	—
9-nitrooleate	0.655	1.800	—	—	—	—	—	—	—	—
10-nitrooleate	0.342	0.495	—	—	—	—	—	—	—	—

OR, expressed per 1 IQR, increase in metabolite concentration (robust-scaled). More information on the models is given in Supplementary Materials. All p-values are nominal and do not account for variable selection or outcome multiplicity. Details regarding scaling are shown in Supplementary Files 1, 2.

**FIGURE 2:**
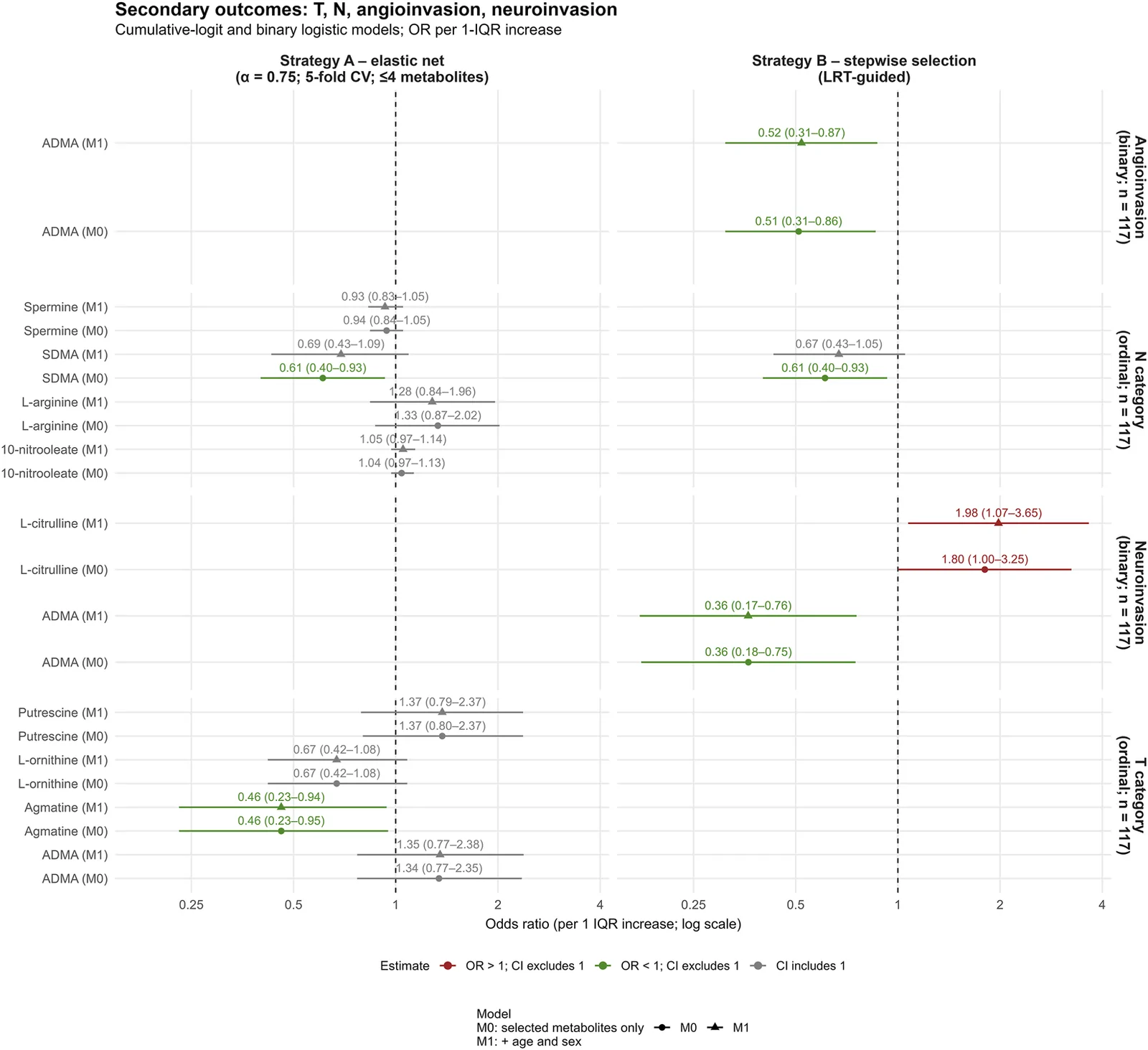
Associations of selected metabolites with secondary tumor outcomes. Forest plots present ORs and 95% CIs for metabolites retained using Strategy A (elastic net; α = 0.75; five-fold cross-validation; one-standard-error rule; maximum of four retained metabolites) or Strategy B (likelihood-ratio-test-guided stepwise selection). T and N categories were analyzed using ordinal regression, whereas angioinvasion and neuroinvasion were analyzed using binary logistic regression. M0 included the retained metabolites; M1 was additionally adjusted for age and sex. ORs are reported per one-interquartile-range increase in metabolite concentration after robust scaling. All displayed T-category, N-category, angioinvasion, and neuroinvasion models included 117 participants. CI, confidence interval; CV, cross-validation; IQR, interquartile range; LRT, likelihood-ratio test.

### 3.2 An additional sensitivity analysis stemming from violation of the proportional odds assumption in case of modelling ordinal outcomes

The proportional odds assumption was assessed using Brant tests, as reported in the Supplementary Materials. Evidence of non-proportionality was consistently observed only for SDMA in models of ordinal nodal involvement. Therefore, partial proportional odds models (PPOMs) were fitted as a fixed-model sensitivity analysis for this outcome (Table 4), with SDMA allowed to have threshold-specific effects and all remaining predictors constrained to satisfy the proportional odds assumption.

**TABLE 4:** Sensitivity analysis regarding the association between SDMA with the N outcome in used modelling strategies—with the use of PPOMs.

Strategy	Model	Cumulative threshold	N	SDMA OR per IQR (bootstrap 95% CI)
A	M0	N1–N2 versus N0	117	0.561 (0.289–0.785)
A	M0	N2 versus N0–N1	117	0.973 (0.581–3.497)
A	M1	N1–N2 versus N0	117	0.638 (0.309–0.954)
A	M1	N2 versus N0–N1	117	1.119 (0.633–4.578)
B	M0	N1–N2 versus N0	117	0.563 (0.308–0.811)
B	M0	N2 versus N0–N1	117	0.953 (0.612–3.378)
B	M1	N1–N2 versus N0	117	0.619 (0.331–0.918)
B	M1	N2 versus N0–N1	117	1.055 (0.622–4.428)

ORs, are reported per one-IQR, increase in SDMA. SDMA, was modelled as a threshold-specific nominal effect, whereas the remaining predictors were constrained to proportional odds. CIs, are percentile intervals derived from 2,000 non-parametric case-bootstrap samples. All bootstrap model fits were valid and produced no numerical warnings.

Likelihood-ratio tests consistently favored the PPOMs over the corresponding proportional odds models. Allowing the association of SDMA to vary between cumulative logit thresholds improved model fit in Strategy A (M0: likelihood-ratio χ^2^(1) = 5.25, p = 0.022; M1: χ^2^(1) = 5.42, p = 0.020) and Strategy B (M0: χ^2^(1) = 5.04, p = 0.025; M1: χ^2^(1) = 5.14, p = 0.023).

The threshold-specific estimates showed an inverse association between higher SDMA concentrations and the odds of nodal involvement at the first cumulative threshold, comparing N1–N2 with N0. The ORs ranged from 0.56 to 0.64 across the four model specifications, and the bootstrap CIs did not include unity. In contrast, no clear association was observed at the second cumulative threshold, comparing N2 with N0–N1. For this contrast, the ORs were close to unity and the bootstrap CIs were wide.

A similar first-threshold pattern was observed under both modelling strategies and in both the M0 and M1 specifications, although the estimates were subsequently attenuated after additional adjustment for serum creatinine, as reported in Section 3.4. Percentile CIs were estimated using 2,000 non-parametric case-bootstrap samples. All bootstrap samples produced valid model fits without numerical warnings for each model specification.

### 3.3 Data completeness and model support

The initial clinical cohort comprised 122 participants. Complete measurements for the panel of 12 candidate metabolites were available for 117 participants (95%), whereas no candidate-metabolite measurements were available for the remaining six participants (4%). No participants had a partially available metabolite panel. The final analytical cohort therefore comprised 117 participants.

Within the analytical cohort, complete data were available for all candidate metabolites, study outcomes, age, and sex. Unless otherwise specified, all metabolite-effect models were fitted using the same 117 participants, with no additional model-specific exclusions. When no metabolite was retained, the corresponding intercept-only or covariate-only fallback specification could involve a different analysis sample size, as documented in the relevant supplementary analyses. The effective predictor degrees of freedom were identical to the nominal degrees of freedom in all model specifications, and no rank deficiency was identified.

For binary outcomes, the smaller outcome group contained between 30 and 57 participants. The number of observations in the smaller outcome group per effective predictor degree of freedom ranged from 7.5 to 57 across the reported models. All binary models containing predictors, except the age- and sex-adjusted Strategy B neuroinvasion model, had at least 10 observations in the smaller group per effective predictor degree of freedom. The neuroinvasion model included ADMA, citrulline, age, and sex and had 30 observations in the smaller outcome group for four effective predictor degrees of freedom, corresponding to a descriptive ratio of 7.5. This more limited data support should be considered when interpreting the precision and stability of its estimates. For ordinal outcomes, some cumulative splits were supported by fewer observations, particularly those involving the extreme T, TNM, and N categories. These descriptive sparsity indicators should be considered when interpreting estimates from the more complex models. Detailed variable-availability summaries, outcome counts, model-specific sample sizes, event frequencies, predictor degrees of freedom, and cumulative ordinal split counts are provided in Supplementary File 4.

### 3.4 Creatinine-adjusted sensitivity analysis

Serum creatinine measurements were available for all 117 participants. The median serum creatinine concentration was 0.84 mg/dL (IQR, 0.73–0.96 mg/dL; range, 0.50–2.57 mg/dL). SDMA was moderately positively correlated with serum creatinine (Spearman’s ρ = 0.432; bootstrap 95% CI, 0.263–0.574; p < 0.001), whereas no clear correlation was observed between ADMA and creatinine (ρ = −0.039; bootstrap 95% CI, −0.219 to 0.140; p = 0.675).

In fixed sensitivity analyses, creatinine was added to the final age- and sex-adjusted models without repeating variable selection (Table 5). Additional adjustment for creatinine attenuated the inverse SDMA associations. For binary advanced TNM stage, the SDMA estimate changed from OR 0.573 (95% CI, 0.351–0.935) to OR 0.739 (95% CI, 0.425–1.287). Associations with ordinal TNM stage and nodal involvement were likewise attenuated, and their creatinine-adjusted CIs included unity.

**TABLE 5:** Creatinine-adjusted sensitivity analyses of the principal SDMA and ADMA associations.

Outcome and metabolite	Strategy	Model or cumulative contrast	Reference model reported in Tables 1–3, OR (95% CI)	Age-, sex-, and creatinine-adjusted model, OR (95% CI)
Ordinal TNM stage — SDMA	A	Proportional odds	0.76 (0.51–1.12)	0.846 (0.536–1.336)
Ordinal TNM stage — SDMA	B	Proportional odds	0.71 (0.48–1.05)	0.768 (0.493–1.198)
TNM stage ≥3 — SDMA	B	Binary logistic	0.57 (0.35–0.94)	0.739 (0.425–1.287)
Ordinal N category — SDMA	A	Proportional odds	0.69 (0.43–1.09)	0.819 (0.478–1.405)
Ordinal N category — SDMA	B	Proportional odds	0.67 (0.43–1.05)	0.772 (0.460–1.296)
Ordinal N category — SDMA	A	PPOM: N1–N2 versus N0	0.638 (0.309–0.954)	0.759 (0.346–1.202)
Ordinal N category — SDMA	A	PPOM: N2 versus N0–N1	1.119 (0.633–4.578)	1.338 (0.665–6.835)
Ordinal N category — SDMA	B	PPOM: N1–N2 versus N0	0.619 (0.331–0.918)	0.714 (0.355–1.147)
Ordinal N category — SDMA	B	PPOM: N2 versus N0–N1	1.055 (0.622–4.428)	1.223 (0.698–5.374)
Angioinvasion — ADMA	B	Binary logistic	0.52 (0.31–0.87)	0.516 (0.307–0.866)
Neuroinvasion — ADMA	B	Binary logistic	0.36 (0.17–0.76)	0.354 (0.168–0.746)

All displayed analyses included 117 participants. The reference estimates were reproduced from the corresponding age- and sex-adjusted M1 models reported in Tables 1–3. Creatinine-adjusted sensitivity models added serum creatinine to the corresponding fixed M1 specification without repeating variable selection. For neuroinvasion under Strategy B, the fixed M1 specification included ADMA, citrulline, age, and sex before serum creatinine was added. ORs, for SDMA, ADMA, and creatinine were expressed per one-interquartile-range increase using scaling constants calculated in the complete analytical cohort. CIs, for the proportional-odds and binary logistic sensitivity models were Wald intervals. CIs, for the PPOMs, were percentile intervals obtained from 2,000 non-parametric case-bootstrap samples. All 2,000 bootstrap fits of the creatinine-adjusted PPOMs, were valid and produced no numerical warnings. In the partial proportional odds models, SDMA, was specified as a threshold-specific effect, whereas creatinine and the remaining predictors were constrained to proportional odds. PPOM.

In the partial proportional odds analyses, the inverse association of SDMA with N1–N2 versus N0 was also attenuated after adjustment for creatinine. The creatinine-adjusted estimates were OR 0.759 (bootstrap 95% CI, 0.346–1.202) for Strategy A and OR 0.714 (bootstrap 95% CI, 0.355–1.147) for Strategy B. No clear association was observed for the N2 versus N0–N1 threshold either before or after creatinine adjustment.

By contrast, the ADMA estimates for angioinvasion and neuroinvasion were essentially unchanged after creatinine was added to the corresponding fixed models. For neuroinvasion, the fixed Strategy B model also included citrulline. The age- and sex-adjusted ADMA estimate changed only slightly after additional adjustment for creatinine, from OR 0.362 (95% CI, 0.174–0.756) to OR 0.354 (95% CI, 0.168–0.746).

Overall, the inverse SDMA associations were substantially attenuated after adjustment for serum creatinine, and all fully adjusted CIs included unity. These findings indicate that the apparent relationship between circulating SDMA and pathological stage was sensitive to adjustment for serum creatinine and do not provide clear evidence of a stage-associated association independent of this renal-function-related marker. The ADMA associations with angioinvasion and neuroinvasion were not materially affected. Complete results for the original and creatinine-adjusted M0 and M1 specifications are provided in Supplementary File 5.

### 3.5 Post-hoc sensitivity analysis without merging T0 with T1 as one category (single-record T0 exclusion)

A *post hoc* sensitivity analysis excluding the single T0 observation yielded the same final predictor set under Strategy A—agmatine, putrescine, ornithine, and ADMA—whereas Strategy B again retained no metabolites. The age- and sex-adjusted agmatine estimate was similar to that obtained in the primary analysis (OR per IQR 0.491, 95% CI 0.240–1.004; nominal p = 0.051, compared with OR 0.464, 95% CI 0.228–0.945; nominal p = 0.034). Thus, the direction and magnitude of the association were largely unchanged, although its nominal statistical significance was sensitive to the exclusion of this single observation. Full results are shown in Supplementary File 6.

### 3.6 An additional sensitivity analysis regarding the neuroinvasion outcome

Given the more limited data support for the age- and sex-adjusted Strategy B neuroinvasion model, the fixed neuroinvasion specifications were additionally refitted using Firth bias-reduced logistic regression. The Firth estimates closely agreed with the ordinary maximum-likelihood estimates. In the Strategy B M1 model, the Firth OR was 0.39 (95% profile penalized-likelihood CI, 0.18–0.76) for ADMA and 1.90 (95% CI, 1.06–3.50) for citrulline, compared with ordinary maximum-likelihood estimates of 0.36 (95% CI, 0.17–0.76) and 1.98 (95% CI, 1.07–3.65), respectively. Similar agreement was observed for the metabolite-only M0 specification. No numerical warnings, non-finite estimates, or rank deficiencies were identified. Strategy A had retained no metabolites for neuroinvasion; its intercept-only and age- and sex-adjusted specifications were nevertheless included for completeness. Full paired comparisons of the ordinary and Firth estimates are provided in Supplementary File 7.

## 4 Discussion

This exploratory within-CRC analysis initially identified SDMA as the metabolite most frequently retained across the two modelling strategies, with higher concentrations associated with lower TNM stage and less advanced nodal involvement in metabolite-only models. However, these associations attenuated after adjustment for age and sex and were further weakened after additional adjustment for serum creatinine. Importantly, all CIs in the fully adjusted models included unity. SDMA was also moderately correlated with serum creatinine, supporting renal function as an important determinant of circulating SDMA concentrations. Accordingly, our data do not support SDMA as an independent or convincing stage-associated metabolic signal in CRC. Rather, the initial associations appear to be substantially influenced by renal function and should be regarded as hypothesis-generating. The threshold-specific association observed for N1–N2 versus N0 in the PPOMs was similarly attenuated after creatinine adjustment and therefore does not alter this interpretation.

Nevertheless, altered SDMA metabolism has previously been reported in CRC, although the relationship between circulating free SDMA and tumor-specific methylation pathways remains uncertain. Our study does not provide information on metabolite levels in healthy subjects; however, a previous study showed that serum SDMA concentrations were elevated in patients with CRC compared with healthy controls (Bednarz-Misa et al., 2020). Tissue studies have also reported increased SDMA accumulation in CRC and adjacent colon tissue compared with healthy colon tissue (Brankovic et al., 2017; Manna et al., 2014; Lu et al., 2013; Krzystek-Korpacka et al., 2020).

Although protein symmetric dimethylation and protein arginine methyltransferase 5 (PRMT5) -dependent pathways have been implicated in cancer biology, circulating free SDMA should not be interpreted as a direct surrogate of tumor-specific PRMT5 activity or protein arginine methylation. The present study did not assess PRMT5 activity, protein methylation, methylthioadenosine phosphorylase (MTAP) status, methionine adenosyltransferase 2A (MAT2A) activity, or S-adenosylmethionine (SAM) and methylthioadenosine (MTA) metabolism. Therefore, these mechanisms cannot be inferred from our data and are mentioned only as broader biological context.

In exploratory Strategy B models, higher ADMA concentrations were associated with lower odds of angioinvasion and neuroinvasion. However, ADMA was not retained under Strategy A, and these findings should therefore be considered strategy-dependent exploratory associations rather than evidence of an independent or protective effect of ADMA on invasive CRC behavior. Although the estimates were not materially altered after adjustment for serum creatinine, this does not establish robustness across modelling strategies or biological causality. As a factor inhibiting NO synthesis and therefore impairing angiogenesis, ADMA was evaluated in a study on the treatment effect of bevacizumab in metastatic CRC. It was found that patients with high baseline ADMA level respond worse to the bevacizumab therapy (Dirican et al., 2014). In cell lines study on LoVo cells, the introduction of ADMA attenuated serum starvation-induced apoptosis and significantly restored transcriptomic changes (Li et al., 2013; Zheng et al., 2016). Interestingly, among patients surgically treated with CRC, ADMA serum levels measured preoperatively and postoperatively showed promising accuracy in predicting post-op complications (Bednarz-Misa et al., 2020). To the best of our knowledge, current literature does not provide data of the correlation between angioinvasion, neuroinvasion in the tumor and ADMA serum levels.

Under Strategy A, lower circulating agmatine concentrations were associated with a higher T category. Because agmatine was not retained under Strategy B, this finding should be regarded as a strategy-dependent exploratory association. The apparently divergent literature on agmatine may, at least in part, reflect differences in the biological compartment being studied. In human CRC, agmatine concentrations have been reported to be lower in tumor tissue than in adjacent macroscopically normal tissue (Molderings et al., 2004), while experimental exposure of colorectal cancer cell lines to exogenous agmatine produced antiproliferative and anti-invasive effects (Tanoglu et al., 2026; Coradduzza et al., 2022). In plasma, lower agmatine concentrations have also been reported in patients with CRC compared with healthy individuals, although no significant difference was observed between early and advanced disease stages (Tanoglu et al., 2026; Coradduzza et al., 2022). By contrast, experimental studies of gut microbiota-derived agmatine have shown that locally produced luminal agmatine can promote intestinal inflammation and activate Wnt/β-catenin signaling, thereby enhancing colorectal tumorigenesis in animal models (Lu et al., 2024; Liu et al., 2018). These observations should not be considered directly contradictory, because circulating agmatine, intracellular or tumor-tissue agmatine, and microbiota-derived luminal agmatine represent biologically distinct compartments and may be governed by different processes of production, uptake, metabolism, and local exposure. Our study measured serum agmatine only and therefore cannot determine whether the observed association with T category reflects tumor-tissue agmatine metabolism, intestinal microbial production, or systemic redistribution. Accordingly, the present finding should be interpreted as a preliminary circulating metabolic association rather than evidence for a specific local mechanism.

The role of NO_2_-FAs in colorectal cancer remains insufficiently characterized, with most functional insights derived from studies in breast cancer models. Current evidence indicates that NO_2_-FAs can reversibly inhibit the 26S proteasome and Rad51. As potential therapeutic agents, they are considered to carry a low risk of inducing secondary drug resistance. Notably, NO_2_-FAs preferentially trigger cell death in tumor cells while exerting only limited cytotoxic effects on normal cells (Kühn et al., 2018; Roos et al., 2024). We identified no clear association between circulating nitrooleate concentrations and the pathological outcomes examined within the CRC cohort. It is plausible that tissue sample studies could give more insight on NO_2_-FAs importance in CRC.

Although no stage-dependent alterations in circulating 9-nitrooleate and 10-nitrooleate were observed in the present cohort, and lower serum ADMA was associated with angioinvasion and neuroinvasion, these findings should not be interpreted solely as a lack of biological relevance of the arginine–NO–NO_2_-FA axis in CRC progression. Experimental studies have suggested that ADMA may influence immune-cell function within the tumor microenvironment (Li et al., 2025); however, these mechanisms were not assessed in the present study. Circulating ADMA cannot be assumed to reflect dendritic-cell or macrophage activity within tumor tissue. Therefore, the observed inverse associations with angioinvasion and neuroinvasion should be regarded only as exploratory serum-level findings and not as evidence that ADMA exerts a protective effect against invasive CRC behavior. A similar interpretative limitation applies to NO_2_-FAs: these electrophilic lipids are produced under inflammatory and oxidative conditions and rapidly undergo reversible Michael addition reactions with cysteine and histidine residues, resulting in post-translational modification of regulatory proteins and modulation of NF-κB, STAT, PPAR-γ, and Keap1/Nrf2-related signaling (Ni et al., 2024). Consequently, serum concentrations of free nitrooleate isomers may not fully capture tissue-level NO_2_-FA signaling, protein-adduct pools, or local redox-dependent activity. Analytical performance should also be considered when interpreting the negative NO_2_-FA findings. The highest analytical imprecision was confined to the lowest tested concentration of 0.0125 nM, at which intra-day CVs were 23.89% for 9-nitrooleate and 24.49% for 10-nitrooleate, and the inter-day CV for 10-nitrooleate was 24.76%. At 0.25 and 1.0 nM, intra- and inter-day CVs were below 15% for both analytes. Thus, analytical variability near the lower end of the measuring range may have attenuated modest biological differences and reduced statistical sensitivity. This limitation is more relevant to 9-nitrooleate, for which 7.7% of measurements were below the LOQ, whereas all 10-nitrooleate measurements were above the LOQ. Consequently, the absence of clear stage-associated associations should not be interpreted as definitive evidence for the absence of variation in circulating NO_2_-FAs. Future paired serum–tumor studies should therefore quantify both free and protein-adducted NO_2_-FAs together with ADMA/SDMA levels and immune-cell phenotyping to clarify whether the systemic changes observed here correspond to local immunometabolic remodeling in CRC.

Serum metabolomic studies exploring metabolic alterations associated with colorectal cancer have increased in recent years. (Wang et al., 2025; Yi et al., 2025; Song et al., 2023). The present study adds exploratory data on circulating metabolites related to the arginine–NO–NO_2_-FA axis, but the observed associations require independent replication before any potential clinical relevance can be considered.

Several limitations should be considered when interpreting these findings. The study included only patients with CRC and therefore cannot determine whether the observed metabolite concentrations are disease-specific or differ from normal physiology. No independent validation cohort was available, and formal stability-selection frequencies, repeated cross-validation of the final models, permutation testing, and bootstrap optimism correction were not performed. Although Firth refitting yielded closely similar estimates for the fixed neuroinvasion models, this sensitivity analysis addresses small-sample coefficient estimation rather than uncertainty or instability introduced by the preceding variable-selection procedure. The ADMA and citrulline findings therefore remain conditional on the Strategy B stepwise procedure and should not be interpreted as independently validated associations. In addition, no formal false-discovery-rate correction was applied, and the reported p-values and CIs do not account fully for uncertainty introduced by variable selection and outcome multiplicity. Findings retained under only one modelling strategy may therefore reflect procedure-dependent selection. The metabolite sets selected by Strategy A are conditional on the fixed elastic-net mixing parameter (α = 0.75), and alternative penalty mixtures could yield different selected sets. Moreover, the Strategy A findings for T and N categories are conditional on the four-metabolite model-size constraint and could differ under alternative truncation rules. Renal function represents a particularly important limitation for the interpretation of circulating SDMA. Because adjustment for serum creatinine substantially attenuated the SDMA estimates and all fully adjusted CIs included unity, the present data cannot distinguish a tumor-stage-related component of SDMA variability from renal-function-related variation. eGFR was not available in the source clinical dataset and therefore could not be incorporated into the analysis; serum creatinine was used as the available marker of renal function. Future studies should preferably include eGFR or other validated measures of renal filtration when evaluating circulating SDMA. Corresponding tissue-level data, including tumor and adjacent-normal ADMA/SDMA concentrations, PRMT5, MTAP, MAT2A, symmetric dimethylarginine-modified proteins, and NOS/iNOS expression, were not available for the present cohort. Therefore, direct correlations between circulating metabolites and tumor-localized molecular changes could not be assessed. Although serum creatinine was incorporated in an additional sensitivity analysis, other potential determinants of SDMA and ADMA, including inflammation, nutritional status, body composition, comorbidities, and medication use, were not comprehensively available. Potential nonlinear associations were not formally evaluated and cannot be excluded. Moreover, because five of the 122 initially enrolled participants had no available candidate-metabolite measurements, selection bias related to complete-panel availability, likewise, cannot be excluded. The results should consequently be regarded as hypothesis-generating and require confirmation in an independent cohort.

## 5 Conclusion

This exploratory study identified preliminary associations between circulating metabolites of the arginine–NO axis and pathological characteristics within a cohort of patients with CRC. SDMA showed recurrent inverse associations with TNM stage and nodal involvement in the initial models; however, these associations were substantially attenuated after adjustment for age, sex, and serum creatinine, with the fully adjusted CIs including unity. Therefore, the present data do not support SDMA as an independent stage-associated metabolic signal, and its apparent relationship with pathological stage should be considered substantially influenced by renal function and hypothesis-generating. The ADMA associations with angioinvasion and neuroinvasion, the citrulline association with neuroinvasion, and the agmatine association with T category were dependent on the variable-selection strategy. No clear stage-associated pattern was observed for circulating 9-nitrooleate or 10-nitrooleate. These findings do not establish disease-specific biomarkers or molecular mechanisms and require validation in independent cohorts.

## Data Availability

The original contributions presented in the study are included in the article/Supplementary Material, further inquiries can be directed to the corresponding author.
