# Supplementary material for: Circulating arginine–nitric oxide pathway metabolites and nitrated fatty acids in relation to pathological features of colorectal cancer
Source: Front Mol Biosci. 2026 Sep 29;13:1910147. doi: 10.3389/fmolb.2026.1910147 (PMC13657980; doi:10.3389/fmolb.2026.1910147)
Supplement: Supplementary file 1 [file DataSheet1.docx]

**SUPPLEMENTARY MATERIALS – README**

The supplementary materials provide a detailed numerical record of the modelling results reported in the manuscript. They include outputs generated under the two exploratory modelling strategies used in the study, a fixed-model sensitivity analysis addressing violation of the proportional odds assumption for ordinal nodal involvement, a separate audit of data completeness and model support, and a creatinine-adjusted sensitivity analysis of the principal SDMA and ADMA associations.

Together, the supplementary files document model structure, estimates, uncertainty, diagnostics, analytical-cohort definition, renal-function-related sensitivity analyses, and the analytical decisions underlying the reported findings, thereby supporting transparency and reproducibility.

**SUPPLEMENTARY FILE 1: REGULARIZATION-BASED MODELLING RESULTS — STRATEGY A**

This file contains the full set of results obtained using Strategy A, based on penalised variable selection followed by unpenalised model refitting.

**Contents**

- Separate worksheets for each analysed outcome:
  - TNM stage, ordinal
  - TNM ≥3, binary
  - T category, ordinal
  - N category, ordinal
  - Angioinvasion, binary
  - Neuroinvasion, binary
- For each outcome and model, M0 and M1:
  - Metabolites retained after penalised selection
  - Odds ratios with 95% confidence intervals
  - Model comparison statistics, including likelihood-ratio tests where applicable
- For ordinal outcomes:
  - Results of Brant tests assessing the proportional odds assumption

**Modelling framework**

- M0 models include metabolites only.
- M1 models additionally adjust for age and sex.
- Ordinal outcomes are analysed using cumulative logit regression models.
- Binary outcomes are analysed using logistic regression.
- Metabolite effects are expressed per one interquartile-range increase following robust scaling based on the median and IQR.

**Penalised variable selection**

- Penalised regression was used exclusively as a variable-selection step to reduce model dimensionality and limit overfitting in the presence of correlated metabolomic features.
- Elastic-net regularisation was applied, allowing simultaneous coefficient shrinkage and handling of correlated predictors.
- The regularisation parameter was selected by cross-validation using a conservative criterion favouring simpler models.
- To preserve interpretability and enable conventional estimation, all metabolites retained after penalisation were subsequently included in unpenalised regression models, from which the reported effect estimates and confidence intervals were obtained.
- Penalisation was not used for final effect estimation or hypothesis testing, but solely to guide parsimonious model specification.

**SUPPLEMENTARY FILE 2: STEPWISE LIKELIHOOD-BASED MODELLING RESULTS — STRATEGY B**

This file contains the full set of results obtained using Strategy B, based on likelihood-ratio-test-guided stepwise variable selection.

**Contents**

- Separate worksheets for each analysed outcome, mirroring Supplementary File 1.
- For each outcome and model, M0 and M1:
  - Metabolites retained after stepwise selection
  - Odds ratios with 95% confidence intervals
  - Model comparison statistics
- For ordinal outcomes:
  - Results of Brant tests assessing the proportional odds assumption

**Modelling framework**

- Outcome definitions, covariates, and scaling procedures are identical to those used in Strategy A.
- M0 and M1 models are defined analogously to Strategy A.
- Ordinal and binary outcomes are analysed using the same regression frameworks as in Strategy A.

**SUPPLEMENTARY FILE 3: PARTIAL PROPORTIONAL ODDS SENSITIVITY ANALYSIS FOR NODAL INVOLVEMENT**

This file contains the fixed-model sensitivity analysis performed in response to evidence of violation of the proportional odds assumption for SDMA in models of ordinal nodal involvement.

No variable selection was repeated in this analysis. The model specifications correspond directly to the metabolites retained under Strategies A and B in the primary analyses.

**Model specifications**

- Strategy A:
  - M0: SDMA, arginine, spermine, and 10-nitrooleate
  - M1: SDMA, arginine, spermine, 10-nitrooleate, age, and sex
- Strategy B:
  - M0: SDMA
  - M1: SDMA, age, and sex

In all partial proportional odds models, SDMA was allowed to have separate effects across the two cumulative-logit thresholds. All remaining predictors were constrained to satisfy the proportional odds assumption.

**Reported threshold-specific contrasts**

- N1–N2 versus N0
- N2 versus N0–N1

Odds ratios are expressed per one-IQR increase in SDMA concentration, using robust-scaling constants estimated from the analytical cohort before bootstrap resampling.

**Contents**

- Revised threshold-specific SDMA odds ratios for each strategy and model
- Percentile 95% confidence intervals derived from 2,000 non-parametric case-bootstrap samples
- Likelihood-ratio comparisons between the corresponding proportional odds and partial proportional odds models
- Bootstrap fit-success rates and summaries of any unsuccessful model fits
- Diagnostics for the models fitted to the original analytical data
- N-category counts for the analytical cohort
- Explicit model specifications
- Metabolite scaling constants
- Session information documenting the analytical environment

**Numerical estimation and uncertainty**

- The partial proportional odds models were fitted using a cumulative-link implementation in which SDMA was specified as a threshold-specific nominal effect.
- The proportional odds and partial proportional odds models were compared using likelihood-ratio tests.
- Wald confidence intervals from the original partial proportional odds implementation were not used because they were numerically implausible.
- Uncertainty was instead quantified using percentile confidence intervals based on 2,000 non-parametric case-bootstrap samples.
- All 2,000 bootstrap samples produced valid model fits without numerical warnings for each of the four fixed model specifications.

**SUPPLEMENTARY FILE 4: DATA COMPLETENESS, MODEL SAMPLE SIZE, AND MODEL-SUPPORT AUDIT**

This file contains a fixed-model audit of the analytical cohort, variable availability, outcome frequencies, model-specific sample sizes, predictor degrees of freedom, and descriptive indicators of data support for the reported regression models.

The audit does not repeat variable selection and does not refit the models. It uses the final model specifications reported in the manuscript.

**Analytical-cohort definition**

- The initial clinical cohort comprised 122 participants.
- Complete measurements for all 12 candidate metabolites were available for 117 participants, corresponding to 95.9% of the initial cohort.
- No candidate-metabolite measurements were available for five participants, corresponding to 4.1% of the initial cohort.
- No participants had a partially available candidate-metabolite panel.
- The final analytical cohort therefore comprised 117 participants.

Within the analytical cohort, complete data were available for all candidate metabolites, study outcomes, age, and sex. All reported models used the same 117 participants, with no additional model-specific exclusions.

**Contents**

- Summary of the initial and final analytical cohorts
- Availability status of the complete 12-metabolite panel
- Variable-level availability in:
  - the initial clinical cohort
  - the final analytical cohort
- Outcome-category counts and percentages in the analytical cohort
- Explicit final model specifications
- Model-specific sample sizes
- Nominal and effective predictor degrees of freedom
- Total number of estimated model parameters
- Model-matrix rank assessment
- Event and non-event counts for binary outcomes
- Descriptive events-per-predictor-degree-of-freedom calculations
- Counts on both sides of each cumulative threshold for ordinal outcomes
- Descriptive ratios of the smaller cumulative group to the effective predictor degrees of freedom
- Session information documenting the analytical environment

**Binary-outcome model support**

For binary outcomes, the following quantities are reported for each final model specification:

- Number of events
- Number of non-events
- Size of the smaller outcome group
- Effective predictor degrees of freedom
- Events per effective predictor degree of freedom
- Non-events per effective predictor degree of freedom
- Number of observations in the smaller outcome group per effective predictor degree of freedom

The smaller outcome group contained between 30 and 57 participants across the reported binary models. The number of observations in the smaller outcome group per effective predictor degree of freedom ranged from 7.5 to 57 across the reported binary models. The lowest value occurred in the age- and sex-adjusted Strategy B neuroinvasion model, which included ADMA, citrulline, age, and sex.

These quantities are presented as descriptive indicators of model support and are not interpreted as absolute model-validity criteria.

**Ordinal-outcome model support**

There is no direct and universally accepted equivalent of binary events per variable for proportional odds regression. Therefore, the file reports counts on both sides of every cumulative-logit threshold.

For each threshold, the audit provides:

- Outcome levels at or below the threshold
- Outcome levels above the threshold
- Number of observations on each side of the threshold
- Size of the smaller cumulative group
- Effective predictor degrees of freedom
- Number of observations in the smaller cumulative group per effective predictor degree of freedom

Some extreme ordinal categories were less frequently represented, particularly T1, TNM stages 1 and 4, and N2. Consequently, the corresponding cumulative splits provided more limited data support for the more complex Strategy A models.

The cumulative-split ratios are included only as descriptive sparsity indicators and should not be interpreted as formal diagnostic tests or absolute adequacy thresholds.

**Predictor degrees of freedom and rank assessment**

- Predictor degrees of freedom exclude the binary-model intercept and the threshold parameters of ordinal models.
- Factor predictors contribute their observed number of levels minus one degree of freedom.
- Effective predictor degrees of freedom are calculated from the rank of the corresponding model matrix.
- Effective and nominal predictor degrees of freedom were identical in all final model specifications.
- No rank deficiency was identified.

**SUPPLEMENTARY FILE 5: CREATININE-ADJUSTED SENSITIVITY ANALYSIS**

This file contains a fixed-model sensitivity analysis evaluating whether the principal associations involving SDMA and ADMA were sensitive to additional adjustment for serum creatinine.

Serum creatinine was the only available routinely measured marker related to renal function. eGFR was not calculated because the analysis was designed to use the directly measured creatinine concentration and to avoid introducing a derived variable partly determined by age and sex, which were already included in the adjusted M1 models.

Analytical cohort and data linkage

• Serum creatinine measurements were available for all 117 participants in the final analytical cohort.

• Creatinine data were linked to the analytical dataset using the unique participant identifier, lp.

• The external clinical file contained records for a broader group of participants. The merge was therefore performed as a left join from the final analytical cohort, and records not represented in the analytical cohort were not included.

• No duplicated identifiers or unmatched analytical-cohort identifiers were identified.

• All original and creatinine-adjusted models used the same 117 participants.

Creatinine summary and metabolite correlations

Serum creatinine had a median concentration of 0.84 mg/dL, with an interquartile range of 0.73–0.96 mg/dL and a range of 0.50–2.57 mg/dL.

The file reports:

• Spearman correlations of creatinine with SDMA and ADMA

• Percentile 95% confidence intervals derived from 2,000 non-parametric case-bootstrap samples

• Pearson correlations as supplementary descriptive estimates

SDMA was moderately positively correlated with serum creatinine, whereas no clear correlation was observed between ADMA and creatinine.

Fixed-model sensitivity analysis

No variable selection was repeated. Serum creatinine was added to the fixed final specifications of all models containing SDMA or ADMA.

The following models were evaluated:

• SDMA:
o Ordinal TNM stage under Strategies A and B
o Binary advanced TNM stage under Strategy B
o Ordinal nodal involvement under Strategies A and B
o Partial proportional odds models of nodal involvement under Strategies A and B

• ADMA:
o Ordinal T category under Strategy A
o Angioinvasion under Strategy B
o Neuroinvasion under Strategy B

For each outcome, the file includes:

• The estimate from the corresponding original fixed model

• The estimate after additional adjustment for serum creatinine

• Odds ratios and 95% confidence intervals

• Regression coefficients and standard errors

• The absolute change in the target-metabolite coefficient

• The estimated creatinine effect

• Likelihood-ratio comparison of the original and creatinine-adjusted models

• Collinearity diagnostics, including variance inflation factors and condition numbers

• Convergence and numerical diagnostics

Scaling and model specification

• SDMA, ADMA, the remaining metabolites, and creatinine were expressed per one-interquartile-range increase.

• Median and IQR scaling constants were estimated once from the complete analytical cohort before model fitting and bootstrap resampling.

• M0 sensitivity models added creatinine to the corresponding metabolite-only specification.

• M1 sensitivity models added creatinine to the corresponding specification already adjusted for age and sex.

• Age and sex remained on the same scales and were coded identically to the primary analyses.

Partial proportional odds models

For ordinal nodal involvement:

• SDMA remained specified as a threshold-specific nominal effect.

• Creatinine and all remaining predictors were constrained to satisfy the proportional odds assumption.

• Models were fitted using the ordinal::clm cumulative-link framework.

• The earlier VGAM implementation was not used.

• Percentile 95% confidence intervals were derived from 2,000 non-parametric case-bootstrap samples.

• All 2,000 original and creatinine-adjusted bootstrap fits were valid for every model specification and produced no numerical warnings.

Reported threshold-specific contrasts were:

• N1–N2 versus N0

• N2 versus N0–N1

Principal interpretation

Additional adjustment for creatinine attenuated the inverse SDMA estimates, particularly in models already adjusted for age and sex. After creatinine adjustment, the confidence intervals of the principal M1 SDMA estimates included unity.

The inverse ADMA estimates for angioinvasion and neuroinvasion were essentially unchanged after creatinine was added to the models, consistent with the absence of a clear correlation between ADMA and serum creatinine in the analytical cohort.

These findings indicate that the reported SDMA associations were partly sensitive to adjustment for serum creatinine. They should not be interpreted as establishing that SDMA is independent of renal function. Other potential determinants of SDMA and ADMA, including systemic inflammation, nutritional status, body composition, comorbidities, and medication use, were not comprehensively available and remain potential sources of residual confounding.

Relationship between the main tables and Supplementary File 5

The original reference models were refitted within the creatinine-sensitivity-analysis framework to permit direct comparison with the corresponding creatinine-adjusted models. Minor differences between some refitted confidence intervals in Supplementary File 5 and those reported in Tables 1–3 may reflect numerical implementation, rounding, and, for the partial proportional odds models, independent bootstrap resampling. These differences did not affect the direction or interpretation of the estimates.

To maintain consistency across the main manuscript, Table 4 reproduces the original reference estimates reported in Tables 1–3 and presents the newly estimated creatinine-adjusted results alongside them.

Contents

• README describing the analytical rationale and interpretation

• Audit of the creatinine-data merge

• Summary statistics for serum creatinine

• Correlations of creatinine with SDMA and ADMA

• Original and creatinine-adjusted fixed-model results

• Fixed-model convergence and numerical diagnostics

• Collinearity diagnostics

• Original and creatinine-adjusted threshold-specific PPOM estimates

• Creatinine effects in the PPOM specifications

• Likelihood-ratio comparisons of PPOM specifications before and after adding creatinine

• Bootstrap fit-success summaries and details of any unsuccessful fits

• Explicit model specifications

• Scaling constants

• Session information documenting the analytical environment

**SUPPLEMENTARY FILE 6: PAIRED SENSITIVITY ANALYSIS EXCLUDING THE SINGLE T0 OBSERVATION**

This file contains a post hoc sensitivity analysis evaluating whether the ordinal T-category findings were materially affected by combining the single observation originally classified as T0 with T1 in the primary analysis.

Analytical rationale and paired cross-validation design

- The initial clinical cohort contained one participant classified as T0.
- This observation was also present among the 117 participants with complete measurements for all candidate metabolites.
- Exclusion of T0 left 116 participants in the complete-metabolite analytical cohort.
- Before T0 exclusion, the original random-number-generation sequence was recreated and the primary Strategy A selection for T category was reproduced.
- For Strategy A, all 116 participants common to the primary and sensitivity-analysis cohorts retained their original cross-validation-fold assignments. Only the T0 observation was removed from its original fold.
- Strategy B was rerun after excluding T0 using the same entry and removal criteria as in the primary analysis.

**Principal results**

Strategy A retained the same final set of four metabolites before and after exclusion of T0: agmatine, putrescine, ornithine, and ADMA. Strategy B retained no metabolites in either analysis.

The age- and sex-adjusted agmatine estimate remained similar in direction and magnitude after exclusion of T0. The odds ratio per one-IQR increase was 0.464 (95% CI 0.228–0.945; nominal p = 0.034) in the primary analysis and 0.491 (95% CI 0.240–1.004; nominal p = 0.051) after T0 exclusion. Thus, excluding the single T0 observation did not materially alter the final predictor set or the estimated effect size. However, the nominal p-value marginally crossed 0.05, further emphasizing the borderline and exploratory nature of this association.

**Contents**

- Description of the post hoc analysis and paired-fold procedure
- Cohort audit, including the number of T0 observations and sample sizes before and after exclusion
- Participant-level audit of the original cross-validation folds and retention after T0 exclusion
- Comparison of the final metabolite selections under Strategies A and B
- Penalized coefficients and metabolite rankings before application of the four-metabolite cap
- Unadjusted and age- and sex-adjusted proportional-odds model estimates
- Likelihood-ratio comparisons of M0 and M1 models
- Brant-test results for the fitted ordinal models
- Metabolite scaling constants
- Session information documenting the analytical environment

**SUPPLEMENTARY FILE 7: FIRTH NEUROINVASION SENSITIVITY ANALYSIS**

This file contains a post hoc fixed-model sensitivity analysis evaluating whether the neuroinvasion-model coefficients were materially affected by small-sample estimation using ordinary maximum-likelihood logistic regression.

**Analytical design**

- The analysis used the same 117-participant complete-metabolite cohort as the primary analyses, including 30 participants with neuroinvasion and 87 without neuroinvasion.
- No variable selection was repeated.
- The four model specifications were fixed according to the primary analyses:
  - Strategy A M0: intercept only
  - Strategy A M1: age and sex
  - Strategy B M0: ADMA and citrulline
  - Strategy B M1: ADMA, citrulline, age, and sex
- Ordinary maximum-likelihood logistic regression was used as the reference estimator.
- Firth bias-reduced logistic regression using Jeffreys-prior penalized likelihood was used as the sensitivity estimator.
- Profile penalized-likelihood confidence intervals and penalized likelihood-ratio p-values were used for Firth models containing predictors. Wald inference was used only for the intercept-only Strategy A M0 model.

**Principal results**

The Firth estimates for the Strategy B neuroinvasion models closely agreed with the corresponding ordinary maximum-likelihood estimates. In the metabolite-only M0 model, the Firth odds ratios were 0.385 (95% CI, 0.179–0.747; p = 0.0033) for ADMA and 1.759 (95% CI, 1.001–3.183; p = 0.0498) for citrulline. In the age- and sex-adjusted M1 model, the corresponding estimates were 0.393 (95% CI, 0.181–0.757; p = 0.0036) and 1.897 (95% CI, 1.062–3.503; p = 0.0306). The absolute changes in the ADMA and citrulline log-odds coefficients relative to ordinary maximum-likelihood estimation ranged from approximately 4% to 8%.

No numerical warnings, non-finite estimates, or model-matrix rank deficiencies were identified.

**Contents**

- Analytical-cohort audit
- Explicit fixed-model specifications
- Audit reproducing the primary ordinary maximum-likelihood estimates
- Paired comparison of ordinary and Firth coefficient estimates
- Complete coefficient tables for all four specifications
- Confidence-interval and p-value methods
- Model convergence and numerical diagnostics
- Metabolite scaling constants
- Session information documenting the analytical environment

**Interpretation**

The close agreement between ordinary and Firth estimates indicates that the reported Strategy B neuroinvasion coefficients were not materially altered by the choice of small-sample bias-reduced estimation. This analysis does not address uncertainty arising from the original stepwise variable selection, estimate metabolite-selection stability, or constitute predictive validation. The ADMA and citrulline findings therefore remain strategy-dependent and exploratory.

**NOTES ON INTERPRETATION**

- All reported effect estimates correspond directly to fitted regression models and are shown without post hoc filtering or outcome-based thresholding.
- For proportional odds models, odds ratios represent cumulative odds of being classified at or above a given outcome category rather than comparisons between mutually exclusive outcome groups.
- Evidence of violation of the proportional odds assumption was observed only for SDMA in models of ordinal nodal involvement.
- The corresponding fixed-model sensitivity analysis is reported in Supplementary File 3 and Table 3 of the main manuscript.
- In that sensitivity analysis, the association of SDMA differed between the two cumulative thresholds: an inverse association was observed for N1–N2 versus N0, whereas no clear association was observed for N2 versus N0–N1.
- All primary and sensitivity models were based on the same analytical cohort of 117 participants.
- The model-support ratios reported in Supplementary File 4 are descriptive indicators intended to make the available outcome information transparent. They are not used as binary rules for accepting or rejecting individual models.
- The less frequently represented extreme categories of some ordinal outcomes should be considered when interpreting the precision and stability of estimates from the more complex models.
- Given the exploratory nature of metabolomic feature screening and the use of data-driven variable-selection procedures in Strategies A and B, all findings should be interpreted as hypothesis-generating rather than confirmatory.
- A creatinine-adjusted fixed-model sensitivity analysis of the principal SDMA and ADMA associations is reported in Supplementary File 5 and Table 4 of the main manuscript.
- SDMA was moderately positively correlated with serum creatinine, and additional adjustment for creatinine attenuated the principal SDMA estimates. The creatinine-adjusted M1 confidence intervals included unity.
- ADMA was not clearly correlated with serum creatinine, and its associations with angioinvasion and neuroinvasion were essentially unchanged after creatinine adjustment.
- Adjustment for serum creatinine should not be interpreted as complete adjustment for renal function or for all potential determinants of circulating SDMA and ADMA.
- Reference models were refitted in Supplementary File 5 to permit direct comparisons with the creatinine-adjusted specifications. Minor numerical differences from Tables 1–3 did not alter the direction or interpretation of the findings. Table 4 reproduces the original reference estimates from Tables 1–3.
